# Dissecting Abdominal Aortic Aneurysm Is Aggravated by Genetic Inactivation of LIGHT (TNFSF14)

**DOI:** 10.3390/biomedicines9111518

**Published:** 2021-10-22

**Authors:** Andrea Herrero-Cervera, Carla Espinós-Estévez, Susana Martín-Vañó, Alida Taberner-Cortés, María Aguilar-Ballester, Ángela Vinué, Laura Piqueras, Sergio Martínez-Hervás, Herminia González-Navarro

**Affiliations:** 1 INCLIVA, Institute of Health Research, 46010 Valencia, Spain; anhecer@alumni.uv.es (A.H.-C.); susana.martin@uv.es (S.M.-V.); altacor@doctor.upv.es (A.T.-C.); abama4@alumni.uv.es (M.A.-B.); m.angela.vinue@uv.es (Á.V.); laura.piqueras@uv.es (L.P.); sergio.martinez@uv.es (S.M.-H.); 2 Centro Nacional de Investigaciones Cardiovasculares (CNIC), 28029 Madrid, Spain; carla.ee@me.com; 3 Department of Pharmacology, University of Valencia, 46010 Valencia, Spain; 4 CIBER de Diabetes y Enfermedades Metabólicas Asociadas (CIBERDEM), 28029 Madrid, Spain; 5 Endocrinology and Nutrition Service, Clinic Hospital of Valencia, 46010 Valencia, Spain; 6 Department of Medicine, University of Valencia, 46010 Valencia, Spain; 7 Biochemistry and Molecular Biology Department, University of Valencia, 46010 Valencia, Spain

**Keywords:** abdominal aortic aneurysm, TNFSF14/LIGHT, vascular smooth muscle cells

## Abstract

Abdominal aortic aneurysm (AAA), is a complex disorder characterized by vascular vessel wall remodeling. LIGHT (TNFSF14) is a proinflammatory cytokine associated with vascular disease. In the present study, the impact of genetic inactivation of *Light* was investigated in dissecting AAA induced by angiotensin II (AngII) in the Apolipoprotein E-deficient (*Apoe*^−/−^) mice. Studies in aortic human (ah) vascular smooth muscle cells (VSMC) to study potential translation to human pathology were also performed. AngII-treated *Apoe*^−/−^*Light*^−/−^ mice displayed increased abdominal aorta maximum diameter and AAA severity compared with *Apoe*^−/−^ mice. Notably, reduced smooth muscle α-actin+ area and *Acta2* and *Col1a1* gene expression were observed in AAA from *Apoe*^−/−^*Light*^−/−^ mice, suggesting a loss of VSMC contractile phenotype compared with controls. Decreased *Opn* and augmented *Sox9* expression, which are associated with detrimental and non-contractile osteochondrogenic VSMC phenotypes, were also seen in AngII-treated *Apoe*^−/−^*Light*^−/−^ mouse AAA. Consistent with a role of LIGHT preserving VSMC contractile characteristics, LIGHT-treatment of ahVSMCs diminished the expression of *SOX9* and of the pluripotency marker *CKIT*. These effects were partly mediated through lymphotoxin β receptor (LTβR) as the silencing of its gene ablated LIGHT effects on ahVSMCs. These studies suggest a protective role of LIGHT through mechanisms that prevent VSMC trans-differentiation in an LTβR-dependent manner.

## 1. Introduction

Abdominal aortic aneurysm (AAA) is a complex and multifactorial disease, and a major cause of morbidity and mortality in males older than 65 years of age [1]. Aneurysms remain asymptomatic and are detected by abdominal explorations of unrelated causes or when rupture occurs, engaging fatal events. Potential treatments to prevent their rupture include stabilization of the AAA through invasive or endovascular surgery [1].

AAA develops as the luminal diameter of arteries increases [1] and provokes a vascular dilation due to the weakening of the vessel wall. The lesions in AAA are complex and characterized by elastin fragmentation and degeneration, and leukocytes infiltration. Furthermore, during extravascular remodeling, vascular smooth muscle cells (VSMC) undergo phenotypic switching, acquiring different cell-type phenotypes [2].

LIGHT is a cytokine member of the tumour necrosis factor superfamily 14 (TNFSF14), expressed mainly on T cells and monocytes [3]. It is synthetized membrane-bound although it can be cleaved by proteases, both forms being active. LIGHT maintains an efficient innate and adaptive immune response through its receptors: the herpes virus entry mediator (HVEM) receptor and the lymphotoxin β receptor (LTβR). Through LTβR, LIGHT activates both canonical and non-canonical nuclear factor kappa B (NFkB) pathways, exerting important functions in immune response and lymphorganogenesis [3]. On the other hand, through HVEM signaling, LIGHT activates NFĸB and c-Jun N-terminal Kinase (JNK) pathways in T and B cells, promoting cytokine production, cell survival, and proliferation [4]. Notably, LIGHT levels are elevated in coronary disease [5], clinical heart failure [6], and unstable angina [7], while LTβR is increased in human atherosclerosis [8]. In mice, LTβR deficiency reduced atherosclerosis [9], while LIGHT treatment aggravated lesion size [10]. Notwithstanding, specific VSMC *Ltβr*-deficiency in Apolipoprotein E-deficient (*Apoe*^−/−^) mice worsened lesions indicating atheroprotection by LTβR signaling [11,12].

In the present study, the implication of LIGHT in angiotensin II (AngII)-dissecting AAA in *Apoe*^−/−^ mice was investigated. To this end, *Apoe*^−/−^ and *Apoe*^−/−^*Light*^−/−^ mice were treated with AngII or vehicle, and analyzed for the development, or not, of AAA lesions. In addition, the examination of potentially involved molecular mechanisms was also performed through in vivo and in vitro studies.

## 2. Materials and Methods

Details of the experimental protocols can be found in Supplementary Materials and Methods online.

### 2.1. Study Approval and Mouse Models

All mice used in this study were housed in the animal facilities of the Faculty of Medicine, University of Valencia/INCLIVA. The studies were carried out in male *Apoe*^−/−^ mice (Charles River, Lyon, France), and *Apoe*^−/−^*Light*^−/−^ mice (all on a C57BL/6J background) generated by crossings between an *Apoe*^−/−^ and *Light*^−/−^ (kindly provided by Prof. K. Pfeffer, University of Dusseldorf). Mice had free access to water, were under controlled temperature and humidity conditions (22 ± 2 °C, 55 ± 10%) and with 12 h light-dark cycles (8:00–20:00 h) in a conventional animal facility. All mice were fed with a regular chow diet (Teklad Global Rodent Diets 6.5% fat; Tekland, Envigo, Barcelona, Spain) during the whole experimental procedure. Dissecting AAA as an experimental approach for human aneurysm was induced with AngII-infusion as previously described by Daugherty et al. [13] and as reported by us previously [14]. To this end, osmotic mini-pumps (Alzet, Model 2004, Charles River, Durect, Cupertino, CA, USA) were implanted subcutaneously in twelve-weeks old *Apoe*^−/−^ and *Apoe*^−/−^*Light*^−/−^ male mice under inhaled anaesthesia (5% isoflurane, at the beginning and then 2% isoflurane) and analgesia (0.2 mg/Kg of body weight) to administer either saline (*n* = 12 *Apoe*^−/−^ and *n* = 11 *Apoe*^−/−^*Light*^−/−^) or AngII (Calbiochem^®^; Millipore, Billerica, MA USA) at a dose of 1 µg·kg^−1^·min^−1^ for 28 days (*n* = 29 *Apoe*^−/−^ and *n* = 29 *Apoe*^−/−^*Light*^−/−^), which was the end-point of all experimental mice. The sample size (*n* = 81) was calculated using a priori test with previous published data [15] using the GPower 3.1 program, increasing the number of mice to meet the needs for RNA expression studies in the thoracic aorta with aneurysm. Mice were randomly assigned to the treatments by experienced personnel from the animal care facilities. Mouse mortality during the procedure was due to aortic rupture and these animals were excluded from histological analysis, but they were used for the mortality curve data. At the end of the study, mice were euthanized by cervical dislocation and analyzed as indicated. All animal procedures were approved by the Animal Ethics Committee of INCLIVA (protocol number 2016/VSC/PEA/00032), followed the humane end-points criteria, and followed the 2010/63/EU directive from the European Parliament.

### 2.2. Plasma Determinations

Analysis included triacylglycerol, total cholesterol, apoB-cholesterol and HDL-cholesterol (WAKO, Zaragoza, Spain) measurements as described previously [15]. Samples that failed for HDL precipitation were excluded from the analysis. Cytokine plasma levels were analyzed by using DuoSet^®^ ELISA Development Systems (Minneapolis, MN, USA).

### 2.3. Blood Pressure Measurements

The effectiveness of the AngII was confirmed by the measurement of the systolic (SBP) and diastolic blood pressure (DBP) at weeks 0 and 4 of the treatment, in conscious restrained mice on a warming platform with constant temperature (32–35 °C), using a non-invasive tail-cuff system (CODA; Kent Scientific, Torrington, CT, USA) [16]. For a subgroup of AngII-infused mice, blood pressure was also recorded on a weekly basis. 

### 2.4. Aneurysm Measurements and Classification

At the end of the experimental procedure, quantification of the aneurysm was evaluated ex vivo using a stereoscope (DMD108 Digital Microimaging Device; Leica^®^ Microsystems, Wetzlar, Germany) as previously described [14]. For quantitative analysis of the enlarged vessel identified as aneurysm, five external diameters of the suprarenal aorta of mice were measured. Measurements were at the same distance from the iliac arteries and at the same intervals along the length of the vessel enlargement identified as aneurysm. Aneurysm severity was evaluated based on previous studies [14,16,17,18] by two independent researchers blinded to genotype.

### 2.5. Circulating Leukocyte Populations Analysis

The characterization of the circulating leukocytes was performed by flow cytometry using four different protocols to detect lymphocyte and monocyte subsets, as previously described [15]. To this end, 10 to 50 μL of heparinized whole blood were incubated with the different antibody cocktails according to manufacturer’s recommendations, followed by incubation with FACS lysing solution (BD Biosciences). For analysis accuracy, samples with inefficient erythrocyte lysis were excluded from the study. The leukocyte subset determination was performed using Fortessa Flow cytometer (BD LSR Fortessa^®^ Xnd 20; BD Biosciences, Franklin Lakes, NJ, USA) and BD FACSDiva™ software.

### 2.6. Histological Characterization of Aneurysmal Lesions and Stainings

Collagen, elastic fibers, acid mucopolysaccharides and calcium deposits, and elastic fibers were stained by Masson trichrome Goldner, Weigert Van Gieson, Alcian blue and Von Kossa, respectively. Elastic fibers integrity, breaks and degradation were graded following standard criteria [18]. For macrophage, VSMC, MMP2, MMP9, and SOX9 content in lesions, sections were incubated with antibodies and treatments as previously described [19]. Immunofluorescences for CD3 determinations were performed as described [20]. Slides were mounted and images were captured with a microscope with a built-in camera, Leica^®^ DMD108 Digital Microimaging Device (Leica Microsystems, Wetzlar, Germany) and with a confocal microscope (TCS, SP8, Leica, Wetzlar, Germany), and were analyzed with Image J/FIJI software. Content of markers was expressed as percentage relative to media area, to extravascular tissue area or as number of cells per area unit.

### 2.7. Aortic Human VSMC Cell Culture Experiments

Aortic human (ah) VSMCs were commercially obtained and cultured in 231 medium and smooth muscle growth factor as previously described [20]. LIGHT and AngII overnight treatments were performed in 70% confluent cells or in 72 h pre-treated cells with siRNA Control or siRNA LTBR. VSMCs were cultured in six well plates and were randomly assigned to the different treatments. After treatments, cells were harvested for gene expression analysis by qPCR (Supplementary Materials and Methods online).

### 2.8. Gene Expression Analysis by Quantitative Real-Time qPCR

Total RNA was obtained from aneurysm mouse tissue or cultured ahVSMCs using TRIzol reagent (Invitrogen, Carlsbad, CA, USA). Between 500 ng and 1 µg of RNA were reverse transcribed with the Maxima First-Strand kit (Fermentas, Waltham, MA, USA). The genes of interest were amplified with Luminaris Color HiGreen High ROX qPCR Master Mix (Fermentas, Waltham, MA, USA) on a 7900 FastSystem thermal cycler and results were analyzed with the formula 2^−ΔΔCt^. mRNA levels were normalized to the *Cyclophilin* gene expression in mouse tissues and to the *GAPDH* mRNA levels in ahVSMCs and relativized to controls. The primer sequences were obtained from the PrimerBank data base (Massachusetts General Hospital, Harvard University) and are listed in Tables S1 and S2.

### 2.9. Statistical Analysis

Quantitative data are presented as the mean ± the standard error of the mean (SEM) and single data points unless otherwise stated. Mouse sample replicates for in vivo data were obtained from individual mice. Immunohistochemical analysis was performed in 11 sections from the different mouse groups. For some stainings, a smaller number of replicates was used due to the limited amount of tissue sections. All inmunohistochemical stainings were included for analysis unless the staining was unreliable for automated imaging color deconvolution measurements. For ahVSMC culture experiments, replicates were obtained from 3-4 samples and were the average of 2–3 experiments. All samples and mice were randomly treated and analyzed by observers blinded to genotype or treatments. Treatments and collection of data were obtained at the same time in order to avoid confounding effects. The exclusion criteria were applied when data was out of range of the standard curve in each experiment, when samples were lost during the experimentation and when the (non-iterative) Grubbs test identified outliers. Statistical tests were applied after the determination of normal distribution (Shapiro–Wilk and D’Agostino–Pearson normality tests) and equality of variances (F test). In experiments with four groups, comparisons were made regardless of the treatments or the genotype, but only relevant differences were discussed. Where two mouse groups were displayed, AngII-treated *Apoe*^−/−^ mice was referred to as the control. Differences were evaluated with unpaired Student’s *t* test, Mann–Whitney U test (for nonparametric distribution), one-way ANOVA followed by Bonferroni multiple comparison test (more than two groups), and two-way ANOVA followed by Tukey’s or Sidak’s post hoc multiple comparison test (two mouse groups and two treatments). Qualitative variables were displayed as bar-graphs and were evaluated by the Chi-square test. Kaplan–Meier survival curves were compared by the log-rank (Mantel–Cox) test. All statistical tests were run in GraphPad Prism^®^ 9.0.0 (GraphPad Prism Software, La Jolla, CA, USA). Differences were considered statistically significant when *p*-values were below 0.05: *, *p* < 0.05; **, *p* < 0.01; ***, *p* < 0.001; ****, *p* < 0.0001.

## 3. Results

### 3.1. Light Deficiency Promotes Severe Dissecting AAA in Apoe^−/−^ Mice

**Figure 1:**
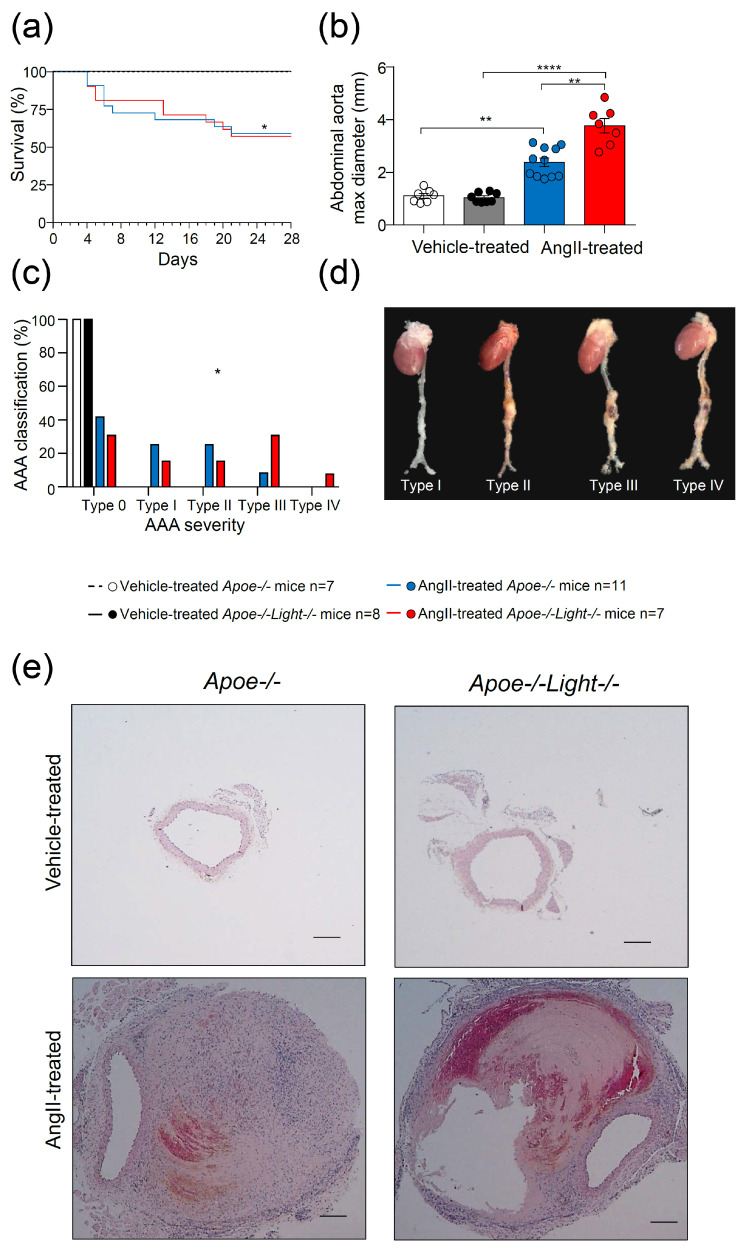
AngII–induced dissecting AAA analysis in *Apoe*^−/−^ and *Apoe*^−/−^*Light*^−/−^ mice. (**a**) Survival curves for vehicle- and AngII-treated mice. (**b**) Comparison of suprarenal abdominal aorta maximal diameter among groups. (**c**) AngII–induced dissecting AAA severity distribution in all four groups of mice. (**d**) Representative images of whole aortas with AngII–induced dissecting AAA classified by severity and (**e**) abdominal aortic cross-sections stained with hematoxylin-eosin. Scale bar: 200 µm. Statistical analysis was: Long-rank (Mantel-Cox) test (**a**), two-way ANOVA followed by Tukey’s multiple comparison test (**b**), and Chi-square test (**c**). * *p* ≤ 0.05, ** *p* < 0.01, and **** *p* < 0.0001.

Body weight (BW) analysis showed no differences among vehicle- and AngII-treated *Apoe*^−/−^ and *Apoe*^−/−^*Light*^−/−^ mice (Supplementary Figure S1a). Weekly monitoring of blood pressure in AngII-treated mice revealed a gradual increase of SBP and DBP in both *Apoe*^−/−^ and *Apoe*^−/−^*Light*^−/−^ mice (Supplementary Figure S1b,c). In AngII-treated *Apoe*^−/−^*Light*^−/−^ mice, SBP was higher at week 1 and 2 (172.6 ± 6,2 and 181.5 ± 9.2 compared with 135.1 ± 7.3 and 146.3 ± 6.3), and DBP at week 2 compared with controls (142.4 ± 9.0 compared with 111.5 ± 5) (Supplementary Figure S1b,c). Circulating plasmatic total- and apoB-cholesterol levels were increased in AngII-treated mice while HDL-cholesterol and triglycerides were similar among the four mice groups (Supplementary Figure S1d–g). Reduced survival times were found in AngII-infused mice compared with vehicle-treated controls, but these were similar between genotypes in both vehicle and AngII treatments (Figure 1a). AngII-treated *Apoe*^−/−^*Light*^−/−^ mice displayed augmented AAA maximum diameter compared with AngII-treated *Apoe*^−/−^ mice (Figure 1b and Supplementary Figure S2a), and these diameters were larger in AngII-treated mice than those in vehicle counterparts. Likewise, AAA severity was higher in AngII-treated *Apoe*^−/−^*Light*^−/−^ mice than in AngII-treated *Apoe*^−/−^ mice, which did not exhibit type IV aneurysms (Figure 1c–e). As expected, vehicle-treated mice did not develop aneurysm (Figure 1c–e). Of note, analysis of lumen/whole vessel area was significantly decreased in AngII-treated mice compared with vehicle-treated mice indicating an increase of the vessel wall during aneurysm development (Supplementary Figure S2b).

### 3.2. Effect of Light Deficiency on Inflammation in Dissecting AAA in Apoe^−/−^ Mice

**Figure 2:**
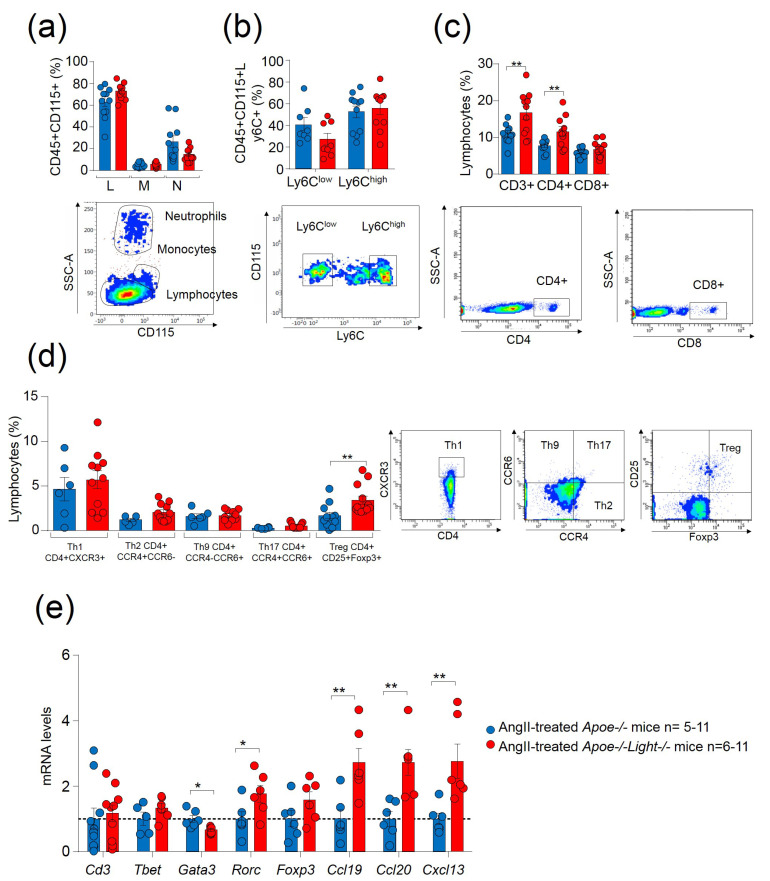
Analysis of inflammation in AngII–induced dissecting AAA in *Apoe*^−/−^ and *Apoe*^−/−^*Light*^−/−^ mice. (**a**) Percentages of circulating lymphocytes, L, monocytes, M, and neutrophils, N, identified in the CD45^+^ blood cell population by morphology and by the CD115 monocyte marker in AngII–treated *Apoe*^−/−^ and *Apoe*^−/−^*Light*^−/−^ mice. (**b**) Percentages of monocyte subsets CD45^+^CD115^+^Ly6C^low^ and CD45^+^CD115^+^Ly6C^hi^ identified within the CD45^+^CD115^+^ population in all groups of mice. (**c**) Circulating levels of CD3^+^, CD4^+^, and CD8^+^ lymphocytes in mice. (**d**) Cellular subpopulations within CD4^+^ T lymphocytes: CD4^+^CXCR3^+^ Th1, CD4^+^CCR4^+^CCR6^−^ Th2, CD4^+^CCR4^−^CCR6^+^ Th9, CD4^+^CCR4^+^CCR6^+^ Th17, and CD4^+^Foxp3^+^CD25^+^ Treg for both mice groups. Representative flow cytometry plots of the gating strategies are displayed. (**e**) mRNA levels of *Cd3*, *Tbet*, *Gata3*, *Rorc* and *Foxp3* genes in aneurysmal tissue from AngII-treated mice. mRNA levels were normalized to *Cyclophilin* mRNA levels and relativized to AngII-treated *Apoe*^−/−^ mice. Statistical analysis was performed using Student’s *t* test (**a**–**e**) and Mann–Whitney U test (in (**d**) for Th17 and Treg cells). * *p* ≤ 0.05 and ** *p* < 0.01.

Further analysis in AngII-treated mice demonstrated that *Light* inactivation did not have an effect in circulating lymphocytes, monocytes, or neutrophils (Figure 2a). Similarly, no differences were observed in Ly6C^low^ and proinflammatory Ly6C^hi^ monocytes between AngII-treated *Apoe*^−/−^*Light*^−/−^ and *Apoe*^−/−^ mice (Figure 2b). In contrast, AngII-treated *Apoe*^−/−^*Light*^−/−^ mice displayed increased CD3^+^ and CD4^+^ T cells compared with AngII-treated *Apoe*^−/−^ mice without changes in CD8+T cells (Figure 2c). Circulating CD4^+^CXCR3^+^ Th1, CD4^+^CCR4^+^CCR6^−^ Th2, CD4^+^CCR4^−^CCR6^+^ Th9, and CD4^+^CCR4^+^CCR196^+^ Th17 remained unchanged between genotypes, but CD25^+^FOXP3^+^ Treg cells were augmented in AngII-treated *Apoe*^−/−^*Light*^−/−^ mice (Figure 2d). Gene expression analysis in AAA tissue revealed no changes in the T cell marker *Cd3* or in the transcription factors *Tbet* and *Foxp3* genes, determinants for Th1 and Treg cells (Figure 2e). However, AngII-treated *Apoe*^−/−^*Light*^−/−^ mice aneurysms showed reduced expression of the Th2 transcription factor *Gata3* gene, and augmented mRNA levels of the Th17 transcription factor *Rorc* gene (Figure 2e). Interestingly, we also found increased mRNA levels of important genes in lymphorganogenesis and tissular local immunity: *Ccl19, Ccl20* and *Cxcl13* (Figure 2e). Circulating inflammatory TNFα levels were augmented in AngII-treated mice compared with vehicle-treated counterparts although IL6 remained unchanged (Supplementary Figure S2c). Cytokine levels were alike between genotypes. Hence, despite the attributed role of LIGHT in the promotion of inflammatory mediators, its inactivation did not ameliorate inflammation.

### 3.3. Light Deficiency Modifies Lesion Characteristics and Gene Expression Pattern in Dissecting AngII-AAA

**Figure 3:**
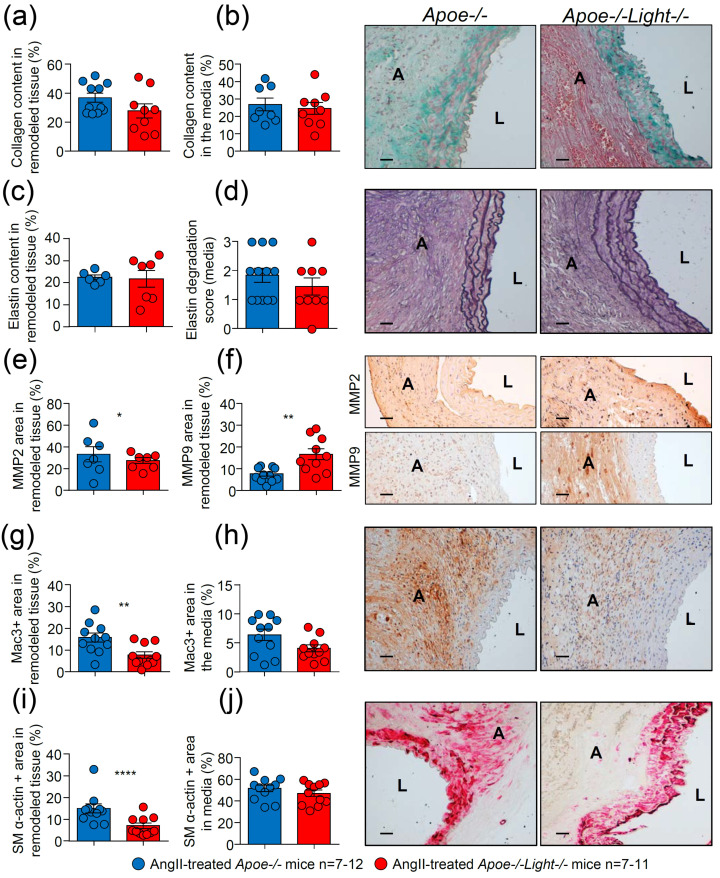
Impact of *Light* deficiency in AngII–induced dissecting AAA characteristics in *Apoe*^−/−^mice. Collagen content in (**a**) the remodeled aneurysm tissue and in (**b**) the media in cross-sections from AngII–treated mice. (**c**) Percentage of elastin in remodeled tissue and (**d**) elastic degradation score in the media in AngII–treated mice. (**e**) MMP2^+^, (**f**) MMP9^+^, (**g**,**h**) Mac3^+^ and (**i**,**j**) SM α-actin^+^ areas as % in the remodeled aneurysm tissue and in the media in abdominal aortic cross-sections of AngII–treated mice. Representative images of the stainings are shown next to the quantifications. Scale bar: 50 µm. L: lumen and A: aneurysm. Data are mean ± SEM. Statistical analysis was: Student’s *t*-test (**a**–**h**,**j**) and Mann–Whitney U test (**i**). * *p* ≤ 0.05, ** *p* < 0.01, and **** *p* < 0.0001.

Aneurysm cross-section analysis revealed similar collagen and elastin contents and elastin degradation score between AngII-treated *Apoe*^−/−^*Light*^−/−^ and *Apoe*^−/−^ mice (Figure 3a–d and Supplementary Figure S3a,b). MMP2 area was decreased while the MMP9 area was augmented in AAA from AngII-treated *Apoe*^−/−^*Light*^−/−^ mice compared with controls (Figure 3e,f and Supplementary Figure S3c,d). AngII-treated *Apoe*^−/−^*Light*^−/−^ mice also showed reduced Mac3^+^ macrophage area in aneurysm, but not in the media, compared with that of AngII-treated *Apoe*^−/−^ mice (Figure 3g,h and Supplementary Figure S3e). Consistent with the above results (Figure 2), Mac3^+^ area was significantly higher in Ang-II treated mice compared with vehicle-treated controls indicating that enhanced inflammation associates with aneurysm formation (Supplementary Figure S2d). Likewise, smooth muscle (SM) α-actin+ area in AngII-treated *Apoe*^−/−^*Light*^−/−^ mice was significantly reduced in the aneurysm, but not in the media, compared with that of AngII-treated *Apoe*^−/−^ mice *(*Figure 3i,j and Supplementary Figure S3f). Analysis of CD3^+^ lymphocyte content in aneurysm was similar regardless of the treatment and genotype (Supplementary Figure S2e). Therefore, *Light* inactivation aggravates aneurysm in *Apoe*^−/−^ mice and changes characteristics of AAA lesions.

**Figure 4:**
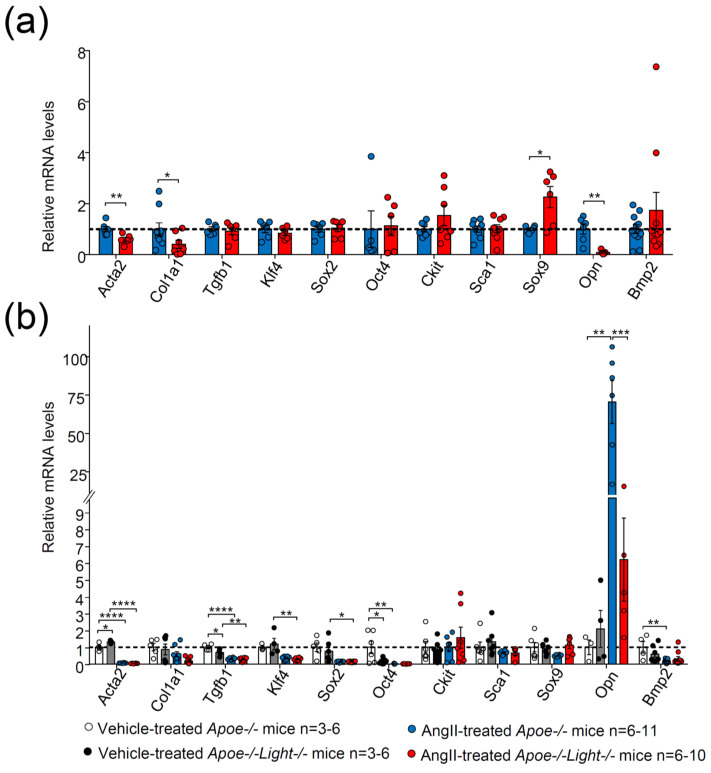
Gene expression analysis in abdominal aortic tissue in vehicle- and AngII–treated *Apoe*^−/−^ and *Apoe*^−/−^*Light*^−/−^ mice. mRNA levels of *Acta2, Col1a1, Tgfb1, Klf4, Sox2, Oct4, Ckit, Sca1, Sox9, Opn*, and *Bmp2* in (**a**) AngII-treated mice and in (**b**) all four mouse groups. mRNA levels were normalized to *Cyclophilin* mRNA levels and relativized to (**a**) AngII-treated Apoe^−/−^ mice or to (**b**) vehicle-treated Apoe^−/−^ mice. Statistical analyses were: (**a**) Student’s *t*-test and Mann–Whitney U test (*Oct4*) and (**b**) Two-way ANOVA followed by Tukey’s multiple comparison test. Data are mean ± SEM. * *p* ≤ 0.05, ** *p* < 0.01 *** *p* < 0.001, and **** *p* < 0.0001.

Diminished SM α-actin^+^ area in *Light*-deficient AAA is compatible with a loss of contractile VSMC phenotype. Hence, the expression of genes associated with VSMC phenotype switching were investigated. AngII-treated *Apoe*^−/−^*Light*^−/−^ mouse AAA displayed decreased mRNA levels of *Acta2*, *Col1a1,* and *Opn* and augmented expression levels of the osteochondrogenic marker *Sox9* without changes in the gene expression of *Tgfb1, Klf4, Sox2*, *Oct4, Ckit*, *Sca*, and *Bmp2* (Figure 4a).

To further explore the gene expression changes during AAA development, comparisons were also made with vehicle-treated mice. Both AngII-treated *Apoe*^−/−^*Light*^−/−^ and *Apoe*^−/−^ mice displayed diminished mRNA levels of *Acta2* and *Tgfb1* genes (Figure 4b) compared with vehicle-treated mice. In addition, AngII-treated *Apoe*^−/−^*Light*^−/−^ mice showed reduced expression of *Klf4* and *Sox2,* while only AngII-treated *Apoe*^−/−^ mice exhibited decreased *Oct4*, *Bmp2*, along with augmented *Opn* mRNA levels compared with vehicle counterparts (Figure 4b). Comparison between the two vehicle-treated mice groups revealed diminished *Tgfb1* and *Oct4* gene expression levels and augmented *Acta2* mRNA levels in *Apoe*^−/−^*Light*^−/−^ mice compared with vehicle-treated *Apoe*^−/−^ mouse controls (Figure 4b).

**Figure 5:**
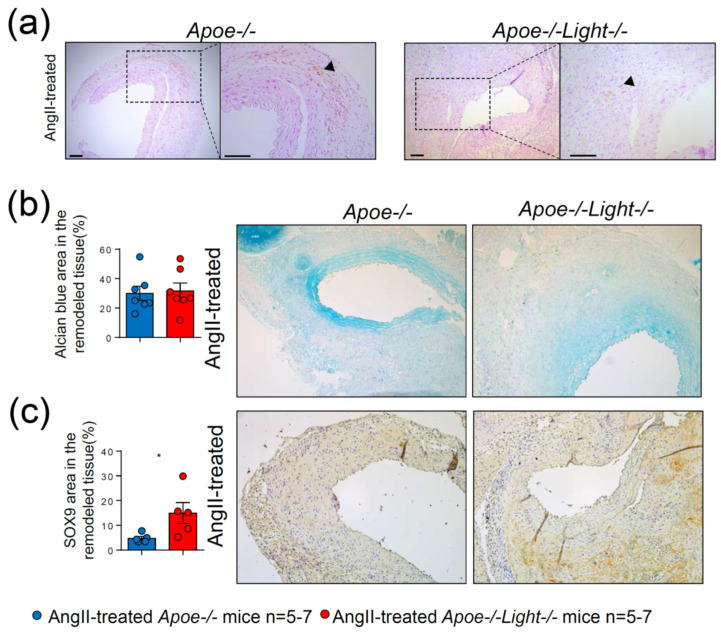
Abdominal aortic cross-section analysis in AngII–treated *Apoe*^−/−^ and *Apoe*^−/−^*Light*^−/−^ mice. (**a**) Images of vascular calcification determined by Von Kossa staining in aortic aneurysm cross-sections in both groups of mice. (**b**) Mucopolysaccharide quantification in aneurysm determined by Alcian Blue staining area in both mouse groups. (**c**) Sox9^+^ area in AngII–treated *Apoe*^−/−^ and *Apoe*^−/−^*Light*^−/−^ mouse aneurysm. Representative images are displayed next to the quantifications. Black triangles point to positive stainings. Scale: 100 µm. Data are mean ± SEM. Statistical analysis was performed using Student’s *t* test. * *p* < 0.05.

Augmented *Sox9* and reduced *Opn* gene expression are associated with aneurysm development, calcification and hyperchondroplasia. Nevertheless, calcification was barely detected in our experimental approach, while the mucopolysaccharide content was alike between AngII-treated *Apoe*^−/−^*Light*^−/−^ and *Apoe*^−/−^ mice (Figure 5a,b). Despite this, SOX9 protein content, which associates with a special trans-differentiation phenotype in vascular injury, was significantly augmented in AAA from *Apoe*^−/−^*Light*^−/−^ mice (Figure 5c), suggesting a role of LIGHT in phenotype switching.

### 3.4. LIGHT-Dependent Signaling Modulates Aortic Human VSMC Phenotype

**Figure 6:**
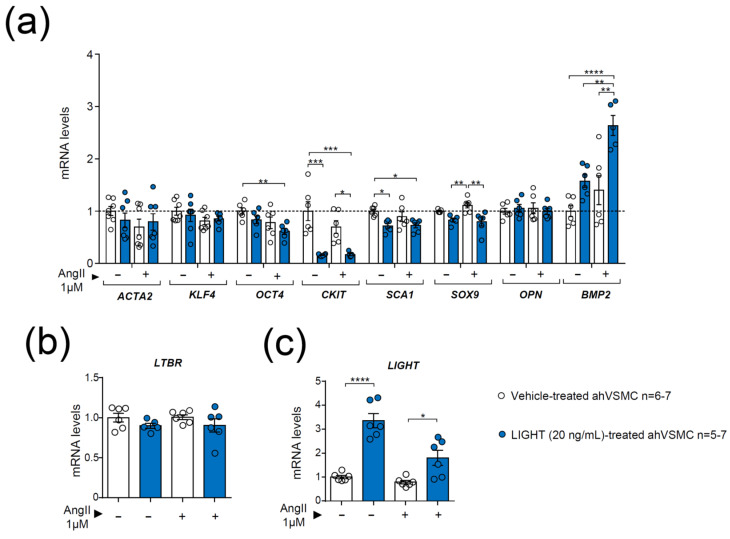
Impact of AngII in the gene expression of vehicle or LIGHT-treated aortic human VSMCs (ahVSMCs). Expression of (**a**) *ACTA2, KLF4, OCT4, CKIT, SCA1, SOX9, OPN*, and *BMP2* mRNA levels in ahVMSCs treated overnight as indicated. mRNA levels of the human (**b**) *LTBR*, (**c**) *LIGHT* genes in ahVSMCs stimulated overnight as indicated. mRNA levels were normalized to *GAPDH* mRNA levels and relativized to vehicle-treated ahVMCs. Data are mean ± SEM. Statistical analysis was performed using two-way ANOVA followed by Tukey’s multiple comparison test. * *p* ≤ 0.05, ** *p* < 0.01, *** *p* < 0.001 and **** *p* < 0.0001.

The effect of LIGHT alone and in combination with AngII to mimic aneurysm conditions was then explored in ahVSMCs. LIGHT treatment of ahVSMCs did not affect *ACTA2*, *KLF4* or *OPN* mRNA levels, regardless of the treatment (Figure 6a). However, LIGHT treatment alone or in combination with AngII reduced *CKIT* and augmented *BMP2* mRNA levels compared with their respective controls without LIGHT (Figure 6a). The gene expression of *SOX9* was also diminished in ahVSMCs treated with a combination of LIGHT and AngII compared with cells treated with AngII alone (Figure 6a). In addition, compared with vehicle-treated ahVSMCs, LIGHT alone reduced *CKIT* and *SCA1* (Figure 6a) gene expression and the combination of LIGHT and AngII diminished *OCT4* while augmented *BMP2* mRNA levels (Figure 6a). Interestingly, LIGHT and AngII-treatment of ahVSMCs increased *LIGHT* mRNA levels, indicating a paracrine effect, but did not affect *LTBR* mRNA levels (Figure 6b,c).

**Figure 7:**
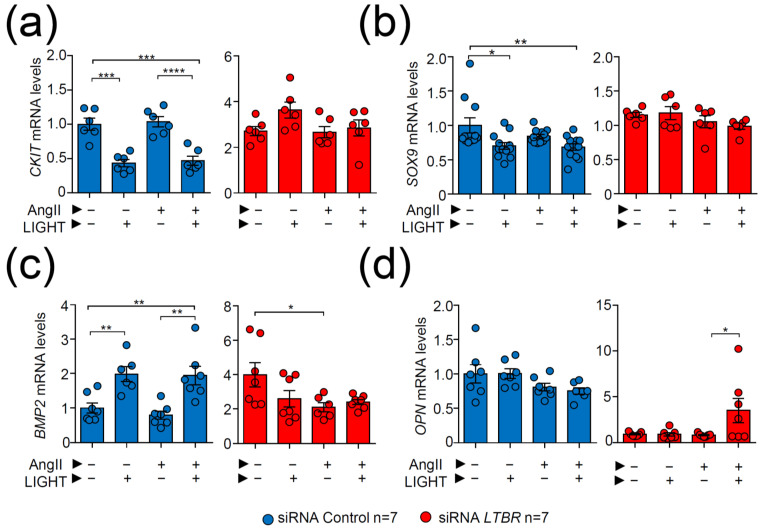
Effect of *LTBR* inactivation in the genetic expression of ahVSMCs treated with AngII and LIGHT. (**a**) *CKIT*, (**b**) *SOX9,* (**c**) *BMP2*, and (**d**) *OPN* mRNA expression levels in ahVSMCs. ahVSMCs were treated with siRNA Control or siRNA *LTBR* and stimulated with vehicle, AngII (1 µM), LIGHT (20 ng), or both AngII and LIGHT. mRNA levels were normalized to *GAPDH* mRNA levels and relativized to vehicle-treated siRNA Control ahVMCs expression. Statistical analysis was performed using two-way ANOVA followed by Tukey’s multiple comparison test. * *p* ≤ 0.05, ** *p* < 0.01, *** *p* < 0.001 and **** *p* < 0.0001.

*LTBR* inactivation through short interference (si) RNA experiments demonstrated that ahVSMC treated with siRNA *LTBR* blunted LIGHT-dependent repression of *CKIT* and *SOX9* genes, unlike treatment with siRNA Control (Figure 7a,b). Likewise, LIGHT-induced expression of *BMP2* was abrogated in siRNA *LTBR*-treated ahVSMCs but not in siRNAControl-treated cells (Figure 7c). Interestingly, *OPN* expression levels were not affected by LIGHT treatment. However, siRNA *LTBR* VSMCs treated with AngII and LIGHT augmented *OPN* gene expression, inversely mirroring what was observed in in vivo AngII-treated *Light*-deficient mice (Figure 7d), probably suggesting a HVEM-mediated effect.

LIGHT treatment modulates the gene expression patterns of ahVSMCs suggesting a potential implication in phenotype switching, most probably toward abnormal osteochondrogenic phenotypes, at least in part, through an LTβR-dependent manner.

## 4. Discussion

The present study demonstrates, for the first time, a protective function of LIGHT against AngII-induced dissecting AAA. Genetic inactivation of *Light* in *Apoe*^−/−^ mice augmented aneurysm maximum diameter and severity compared with *Apoe*^−/−^ mice with intact LIGHT. Further analysis showed decreased SM α-actin^+^ area and mRNA expression levels of *Acta2* and *Col1a1* genes in aneurysms from *Apoe*^−/−^*Light*^−/−^ mice, which are compatible with the loss of VSMC contractile phenotype. Augmented mRNA levels of the *Sox9* gene and diminished *Opn* gene expression, which are both associated with vascular lesion development and osteochondrogenesis, were also observed in *Light*-deficient aneurysm tissue. SOX9 protein content in dissecting AAA of *Apoe*^−/−^*Light*^−/−^ mice was increased, consistent with the acquisition of a special trans-differentiation VSMC phenotype. In fact, LIGHT treatment in ahVSMC provoked a repression of *SOX9*, an upregulation of the osteochondrogenic *BMP2* and *OPN* genes, and diminished the expression of the pluripotency *CKIT* gene. Moreover, *LTBR* gene inactivation through siRNA *LTBR* restored mRNA levels of *CKIT, SOX9*, and *BMP2* genes in LIGHT-treated ahVSMCs which suggested a role of LIGHT/LTβR-dependent signaling in the preservation of VSMCs contractile properties by modulating pluripotency and osteochondrogenic genes. Altogether suggests a protective role of LIGHT in AngII-induced dissecting AAA via an LTβR-dependent mechanism that prevents VSMC trans-differentiation toward detrimental phenotypes associated with vascular vessel wall dysfunction.

Investigations in humans have demonstrated increased LIGHT levels in unstable angina [7] and augmented soluble LTβR in atherosclerosis [8]. Murine investigations have shown a link between LIGHT secretion in NAFLD progression [21] and, consequently, a deficiency in *Light* reduced inflammation and NAFLD [15]. However, others showed aggravated metabolic syndrome in mice deficient in macrophage-derived *Light,* suggesting a protective role of LIGHT [22]. Likewise, LTβR inactivation has been reported to diminish atherosclerosis [9], and LIGHT treatment worsened lesion size [10]. However, specific *Ltβr* deletion in VSMCs exacerbated vascular lesions by a mechanism implicating the alternative LIGHT ligands, lymphotoxin alpha and beta (LTα/LTβ), suggesting atheroprotection through LTβR-dependent signaling [11]. In line with this, our study also points to protection against vascular injury through LTβR-signaling. Notwithstanding, this is the first study to report that this is mediated by LIGHT, and that this axis specifically protects against AAA development and severity.

We acknowledge the limitation that the AAAs in our mouse model develop at the suprarenal abdominal aorta, unlike what usually occurs in humans, where almost all AAAs appear below the renal bifurcations (infrarenal AAAs) [13]. However, AngII infusion generates aneurysms similar to those in humans, characterized by a degeneration of the tunica media and an important leukocyte infiltrate.

VSMC phenotype switching and loss of contractile functions predispose to structural vessel dysfunction and aneurysm and a loss of contractile markers such as *Acta2* [2,23]. A marked decrease in SM α–actin content and *Acta2* and *Col1a1* gene expression in *Light*-deficient AAA is compatible with an accelerated VSMC contractile phenotype loss. A direct effect of LIGHT on *ACTA2* expression or on *COL1A1* (data not shown) gene expression was not seen in ahVSMC in vitro. Likewise, other studies have also reported a lack of upregulation of collagen or SM α-actin in SMCs by direct LIGHT treatment despite, (i) the in vivo effect on lung and skin fibrosis [24] and (ii) the diminished collagen content induced by *Light*-deficiency in vivo in mice during airway remodeling [25]. These results suggest that the in vivo down-regulation of *Acta2* and SM α-actin content could be due to the loss of a potentially protective effect of LIGHT against cellular heterogeneity through phenotype switching.

The increased expression of the *Sox9* marker in *Light*-deficient aneurysms, which has been shown to aggravate vascular lesions [26] and to be determinant in VSMC osteochondrogenic phenotype [27,28], points to a protective role of LIGHT against VSMC trans-differentiation toward detrimental phenotypes. Consistently, ahVSMC treated with human LIGHT repressed *SOX9* mRNA levels while the blocking of LIGHT signaling through *LTBR* gene inactivation restored *SOX9* expression. There is previous experimental evidence indicating that VSMC osteochondrocytic phenotype prevention through LIGHT/LTβR-dependent *Sox9* repression is highly plausible. Thus, (i) *Sox9* expression is mostly limited to VSMC undergoing trans-differentiation processes during vascular lesion development [27] and (ii) *Sox9* repression and prevention of VSMC chondrogenic fate has been observed during development [28] by a NFkB-dependent signaling mechanism, which is the main effector pathway of LIGHT/LTβR axis [29]. Hence, we do believe that it is highly plausible that during vascular remodeling in AAA development, LIGHT/LTβR axis modulate key downstream effectors related to cellular plasticity determinants like SOX9.

Augmented AAA maximum diameter and severity in *Light*-deficient mice also associated with diminished *Opn* gene expression in aneurysm tissue. Despite the known role of OPN in osteochondrogenesis, a chondroid metaplasia due to aberrant cellular phenotypes was previously observed in *Ldlr*^−/−^ mice deficient for *Opn* along with accelerated lesion severity and size [30]. Unlike this last study, we did not observe a correlation between diminished *Opn* expression and chondroid metaplasia, though it was associated with lesion severity. On the other hand, while our in vitro investigations showed that LIGHT treatment of ahVSMC did not affect *OPN* expression in normal conditions, *LTBR*-deficient ahVSMCs treated with AngII and LIGHT enhanced *OPN* levels indicating an upregulation by LIGHT probably in an HVEM-dependent manner. In vitro LIGHT treatment of ahVSMCs also increased *BMP2* mRNA levels in an *LTBR*-dependent manner, a protein also involved in osteochondrogenesis and, on occasion, with opposing SOX9 effects. Altogether suggests that LIGHT-dependent signaling could prevent the severity of lesions by facilitating the maintenance of VSMC contractile phenotype through blocking *SOX9*-mediated changes and by modulating the balance of genes, such as *OPN* and *BMP2*, for a correct physiological and homeostatic osteochondrogenesis.

VSMC phenotype switching in aneurysm and atherosclerosis activates strong changes in important pluripotency genes and phenotype switching. In atherosclerosis, specific *Klf4* deletion in VSMCs prevents lesion development by diminishing VSMC transition to mesenchymatic and foam cell formation [31], while *Oct4* deficiency in VSMCs yields opposing gene expression and large lesions with thin fibrous caps [32]. Moreover, transdifferentiation of VSMC has been shown, through cellular tracing studies to encompass a loss of *Acta2* gene [31,32,33]. In our investigation, neither AAA development in *Light*-deficient mice nor LIGHT treatment in ahVSMCs affected the expression of these major pluripotency genes associated with *Acta2* loss in vascular lesions. However, LIGHT treatment strongly repressed the mRNA expression of the stem cell marker *CKIT* in ahVSMCs. This is an important finding because *CKIT* is up-regulated during intimal hyperplasia through modulation of SMC [33], it is a de-differentiation marker during aortic dilatation in bicuspid aortic valve disease in humans [34] and it is augmented in the cellular membrane of stem cells in human aneurysm [35]. Therefore, inhibition of VSMC pluripotency by LIGHT through de-differentiation gene repression could also contribute to the preservation of the contractile VSMC phenotype.

Our suggested mechanism of LIGHT protection against AAA development through a preservation of contractile VSMC and homeostatic phenotype is not unprecedented. Recent investigations in cancer therapy have demonstrated that LIGHT administered as a fusion protein restored blood vessel homeostasis in tumors in vivo, among others, through increased expression of contractile proteins such as SM α-actin in a LTβR-dependent manner [36]. Altogether, our study and these previously reported data suggest that therapies based in the local delivery of LIGHT in vascular lesions could potentially be useful to stabilize lesions by preserving VSMC phenotype.

In summary, our study is the first to report a possible protective role of LIGHT against vascular injury as in vivo *Light* inactivation aggravates dissecting AAA aneurysm. The diminished expression of contractile markers and the altered osteochondrogenic gene expression pattern also indicate that LIGHT modifies osteochondrogenic VSMC phenotype partly via an LTβR-dependent mechanism. These results suggest a protective role of LIGHT in AAA by preventing VSMC trans-differentiation toward cellular heterogeneity associated with vascular vessel wall dysfunction.

## 5. Conclusions

The present investigation demonstrates that *Light* genetic deficiency aggravates aneurysm development by promoting VSMC switching to detrimental phenotypes associated with *SOX9* expression. Altogether, this suggests a protective role of LIGHT-depending signaling in vascular derangement by preserving VSMC properties.

## Data Availability

All data is available from corresponding author upon reasonable request.

## Supplementary Materials

The following are available online at https://www.mdpi.com/article/10.3390/biomedicines9111518/s1. Supplementary Materials and Methods; Figure S1. Effect of *Light* gene inactivation in metabolic parameters AngII–induced dissecting AAA in *Apoe*^−/−^ mice; Figure S2. Impact of Light gene inactivation in AngII–induced dissecting AAA in Apoe−/− mice; Figure S3. Representative images of stainings in AngII–treated *Apoe*^−/−^ and *Apoe*^−/−^*Light*^−/−^; Table S1: Sequences of the primers used to amplify human genes; Table S2: Sequences of the primers used to amplify murine genes.

## Abbreviations

AAA	Abdominal aortic aneurysm
ah VSMC	Vascular smooth muscle cell
AngII	Angiotensin II
*Apoe* ^−/−^	Apolipoprotein E-deficient
BW	Body weights
DBP	Diastolic blood pressure
HVEM	Herpesvirus Entry Mediator
JNK	c-Jun N-terminal kinase
LTβR	Lymphotoxin-β Receptor
NF-κB	Nuclear factor kappa-light-chain-enhancer of activated B cells
SBP	Systolic blood pressure
SEM	Standard error of the mean
siRNA	Short interference ribonucleic acid
SM	Smooth muscle cell
TNF	Tumor necrosis factor
