# Supplementary material for: Dissecting Abdominal Aortic Aneurysm Is Aggravated by Genetic Inactivation of LIGHT (TNFSF14)
Source: Biomedicines. 2021 Oct 22;9(11):1518. doi: 10.3390/biomedicines9111518 (PMC8615201; doi:10.3390/biomedicines9111518)
Supplement: Supplementary file 1 [file biomedicines-09-01518-s001.zip › biomedicines-1396845-supplementary.pdf]

1. Supplementary Materials and Methods

1.1. Plasma Biochemsitry

Lipid levels were determined in plasma obtained from whole blood of overnight-fasted mice by centrifugation at 12,000 g at 4 °C 30 min in the presence of EDTA. Analysis in plasma included triacylglycerol and total-, apoB- and HDL-cholesterol (WAKO, Zaragoza, Spain). HDL-cholesterol was determined after precipitation of the apolipoprotein B-lipoproteins with dextran sulphate [1].

1.2. Blood Pressure Measurements

Systolic (SBP) and diastolic blood pressure (DBP) were measured using a non-invasive tail-cuff system (CODA; Kent Scientific, Torrington, CT USA) in conscious mice restrained on a warming platform to keep constant temperature (32–35°C) [2]. Mice were acclimatized to the instrument one week before implantation of the pumps to prevent procedure-induced anxiety. During the procedure, individual mice underwent 5 initial pressure readings, which were discarded, and 10 additional cycles of measurements to obtain the actual mean SBP and DBP readings. The criterion for acceptance of data was a standard deviation of <30 mmHg for each session [2]. To avoid variations in blood pressure due to day cycle, all measurements were carried out between 09:00 and 12:00 AM. Two measurement sessions were taken at baseline before the pump implantation and at 28 days of the procedure to ensure the effectivity of the AngII through the increase in blood pressure. In a subset of AngII-infused mice blood pressure was also recorded on a weekly basis.

1.3. Aneurysm Measurements and Classification

At the end of the experimental procedure, quantification of the aneurysm was evaluated using ex vivo using a stereoscope (DMD108 Digital Microimaging Device; Leica® Microsystems, Wetzlar, Germany). For quantitative analysis of the enlarged vessel identified as aneurysm five external diameters of the suprarenal aorta of mice were measured. To avoid variability the five diameter measurements were taken at 5 distances from the iliac bifurcation which was taking as the reference point for measurements for all mice [3,4]. Measurements were at the same distance from the iliac arteries and at the same intervals along the length of the vessel enlargement identified as aneurysm. Aneurysm severity was evaluated macroscopically based on previous studies [5]. AAA were classified as follows: type 0 were those without any aortic dilatation; type I were those with enlarged aorta; type II were those with dilated lumen and frequently with thrombus in the suprarenal aortic region; type III AAA were those with pronounced bulbous form containing thrombus; and type IV were attributed to those with multiple aneurysms with thrombus in suprarenal aorta. Severity and classification were evaluated by two independent investigators blinded to genotypes and treatments.

1.4. Circulating Leukocyte Populations Analysis

The characterization of the circulating leukocytes was performed by flow cytometry. Four different protocols were run to analyze different populations. For monocyte determination 10 μL of heparinized whole blood was incubated for 30 min at room temperature with 1 μL of CD45-FITC, Ly6C-PerCP (553080 and 560525, BD Pharmingen, Franklin Lakes, NJ, USA), and CD115-APC (135509, eBiosciences). Patrolling Ly6C^{low} and proinflammatory Ly6C^{hi} subpopulations of monocytes were determined within the double positive population for the monocyte marker CD115 and the leukocyte CD45 marker. Circulating T lymphocytes were analyzed in 10μl of heparinized blood incubated which were incubated as before with 5 μl of Brilliant stain buffer and with 1 μL of anti-CD3e-APC (BD), anti-CD4-BV421(BD) and anti-CD8a-BV510 (BD). For identification of T helper (Th) cell subsets in mice, the human protocol was adapted for mouse blood as described [6,7]. Briefly, 30 μL of heparinized mouse blood were incubated (30 min, room temperature) with 1 μL of BV421- C-C chemokine receptor (CCR)4 (CD194) (131217, BioLegend), CD4-

FITC (130-102-541, Miltenyi, Bergisch Gladbach, Germany), CXC chemokine receptor (CXCR)3 (CD183)-APC (17-1831-80, eBioscience) and CCR6 (CD196)-PEVio770 (130-103-818, Miltenyi). For regulatory T (Treg) cells, 50 μL of heparinized mouse blood were stained as before with 2.5 μL of anti-CD4 CD4-BV421 (BD) and 5 μL of anti-CD25-APC (Milteny), antibodies followed by 30 min incubation with Foxp3 fixation/permeabilization buffer and a 30 min incubation with 5 μL of anti-Foxp3-PE (Miltenyi). All stains were followed by incubation with FACS lysing solution (BD Biosciences) for 15–20 min for red blood cells lysis and then were analyzed using or Fortessa Flow cytometer (BD LSR Fortessa® Xnd 20; BD Biosciences, Franklin Lakes, NJ USA) in whole blood and analyzed with BD FACSDiva™ software (BD Biosciences, Franklin Lakes, NJ USA) as reported [6].

1.5. Histological Characterization of Aneurysmal Lesions and Stainings

Collagen, elastic fibers, acid mucopolysaccharides, and calcium deposits were stained by Masson trichrome Goldner, Weigert Van Gieson, Alcian blue and Von Kossa methods respectively, following manufacturer's instructions (all from Bio-Optica, Milan, Italy). Slides were mounted with cover slips and Eukitt®, a non-aqueous mounting solution. Elastic fibers' content was expressed as the percentage of elastic fibers area in the media. Elastic fibers' integrity, breaks and degradation was graded after the following criteria [8]: score of 0 for no interruption in the medial elastic lamina, 1 for mild elastin degradation, 2 for noticeable interruption in the medial elastic lamina without change of luminal shape and 3 for long disruptions in the elastic lamina with aortic rupture and bulbous extension of the aortic lumen. For macrophage content, slides were treated for peroxidase inactivation (H_2O_2 3%) and blocking of non-specific binding blocking with horse serum (5%), before being incubated overnight at 4°C with a primary rat anti-Mac3 monoclonal antibody (1/200, sc-19991, Santa Cruz Biotechnology, Santa Cruz, CA, USA), followed by a biotin-conjugated goat anti-rat secondary antibody (1/300, sc-2041, Santa Cruz). Immunodetection was performed with streptavidin-horse radish peroxidase (HRP) (TS-060-HR, LabVision, Fremont, CA, USA) and DAB substrate (SK4100, Vector Laboratories, Burlingame, CA; USA). For MMP9, MMP2 and SOX9 determination, sections were treated for antigen retrieval (citrate buffer 10 mM, pH 6.5 at high pressure and temperature) followed by peroxidase inactivation, blocking, incubation overnight at 4°C with primary antibodies (rabbit anti-MMP-9, 1/200 dilution, [AB19016, UPSTATE-Millipore, Billerica, MA, USA], rabbit anti-MMP2, dilution 1/100 [ab37150,Abcam] antibody, rabbit anti-SOX9, 1/100 dilution, [HPA001758, Sigma St. Louis, MO, USA]), followed by incubation with a biotinylated anti-rabbit secondary antibody (1/500 dilution, sc-2040 Santa Cruz) or followed by incubation with a goat anti-rabbit IgG-HRP (P0448, 1/200 dilution, Dako). Detection was performed as described above with streptavidin-HRP and DAB substrate. Slides were counterstained with hematoxylin and mounted with EUKITT (A10500, Deltalab, Barcelona, Spain). Contractile VSMC determinations were performed by immunostaining of histological sections through incubation at 4°C overnight with the anti-SM α-actin alkaline phosphatase conjugated antibody (1/100 dilution in blocking solution, Sigma). Immunocomplexes were detected using the Fast Red® commercial substrate (Sigma, St. Louis, MO, USA) for alkaline phosphatase and the slides were mounted in glycerol gelatin and sealed. Images were captured with the help of an optical microscope with a built-in camera Leica® DMD108 Digital Microimaging Device (Leica Microsystems, Wetzlar, Germany) and analyzed with the help of the *ImageJ* software (Image Processing and Analysis in Java, NIH). Content of markers was expressed as percentage relative to media area or to extravascular tissue area.

1.6. Human Aortic VSMC Cell Culture Experiments

Primary aortic human (ah)VSMC were obtained commercially and cultured using Medium 231 supplemented with smooth muscle cell growth factor (ThermoFisher Scientific) as described [9]. Cell suspensions were plated on 6-well plates and kept on a humidified 5% CO_2 atmosphere and 20% FBS/DMEM-P/S/A medium until treatments. ahVSMC cultures were used until passage 6-7. For experiments ahVSMC were grown on 6-well

plates to 70-80% of confluency and then treated for 20 h in 0.5% FBS/DMEM-P/S/A medium with vehicle, human soluble LIGHT (20 ng/mL), angiotensin II (AngII, 1 µM) or with a combination of LIGHT (20 ng/mL) and AngII. In silencing experiments, cells were grown as before to 60% of confluency. Cells were then transfected with either human siRNA control, or siRNA *LTBR* (ON-TARGETplus *Smartpool* siRNA, Dharmacon, Cultek S.L.U., Madrid, Spain) using Lipofectamine RNAiMAX (Thermofisher, Massachusetts, USA) for 72 h and then treated for 20 h with human soluble LIGHT (20 ng/mL), AngII (1 µM) or with a combination of LIGHT (20 ng/mL) and AngII (1 µM). After treatments, cells were harvested, and snap-frozen with liquid N_2 and stored for gene expression analysis by qPCR.

2. Supplementary Tables and Figures

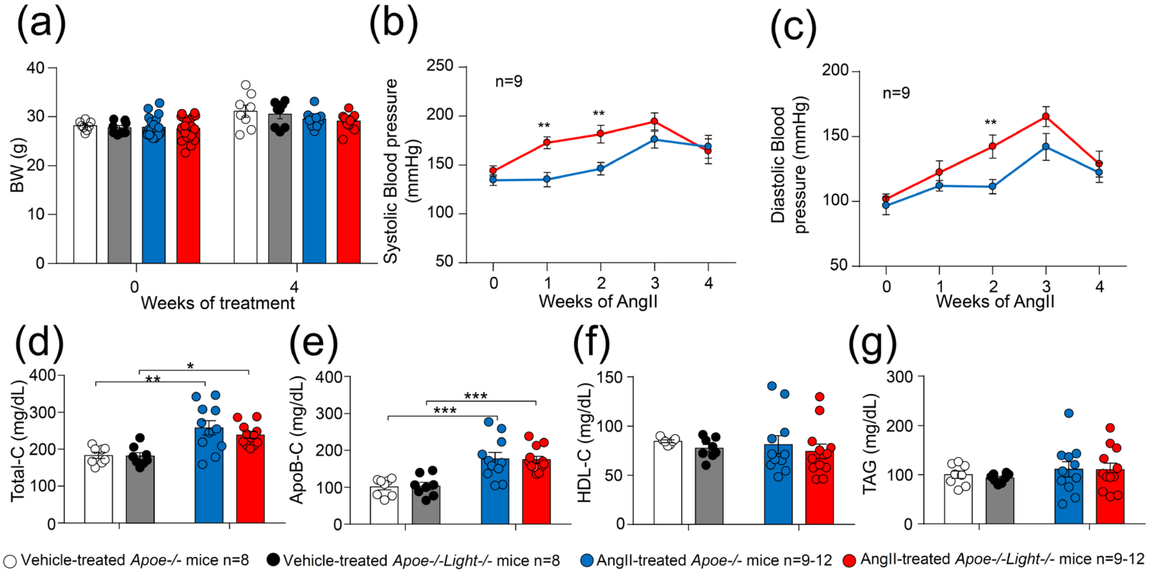

Figure S1. Effect of *Light* gene inactivation in metabolic parameters AngII–induced dissecting AAA in *Apoe*$^{-/-}$ mice. (**a**) Body weight of vehicle-treated and AngII–treated *Apoe*$^{-/-}$ and *Apoe*$^{-/-}$*Light*$^{-/-}$ mice at week 0 and 4 of the treatment. (**b**) Systolic and (**c**) diastolic blood pressure measurements at different time-points during the AngII–treatment in *Apoe*$^{-/-}$ and *Apoe*$^{-/-}$*Light*$^{-/-}$ mice. Plasmatic levels of (d) total cholesterol, (**e**) apoB-cholesterol, (**f**) HDL-cholesterol, and (**g**) triacylglycerol at week 4 of the treatments. SBP: Systolic blood pressure; DBP: Diastolic blood pressure; Total-C: total cholesterol; ApoB-C: Apolipoprotein B cholesterol; HDL-C: HDL-Cholesterol; TAG: Triacylglycerol. Data are shown as bars with individual data points and mean ± SEM. Statistical analysis was done using two-way ANOVA followed by Tukey's multiple comparison test (a and d-g) and multiple comparison student's t-test corrected by the Holm-Sidak method (b,c). *$p \leq 0.05$ and **$p < 0.01$, and ***$p < 0.001$.

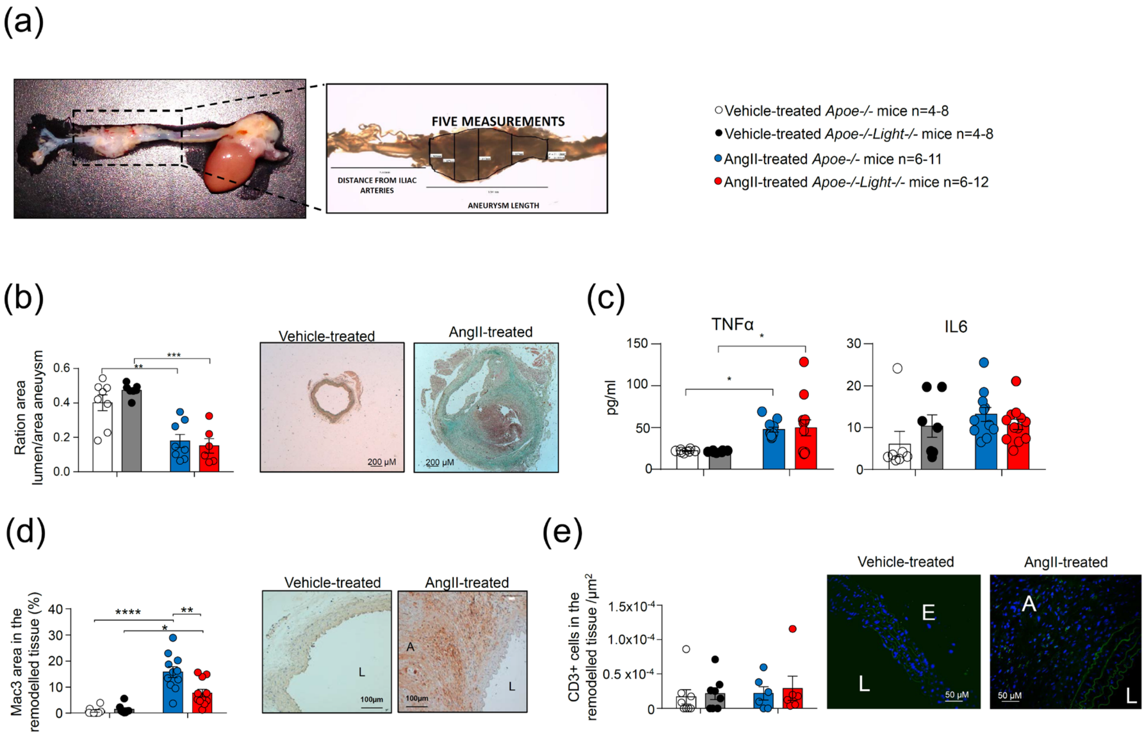

Figure S2. Impact of *Light* gene inactivation in AngII–induced dissecting AAA in *Apoe*$^{-/-}$ mice. (**a**) Representative measurements taken for an artery with aneurysm using a stereoscope and the same artery at higher magnification. (**b**) Lumen area/aneurysm area of vehicle-treated and AngII–treated *Apoe*$^{-/-}$ and *Apoe*$^{-/-}$*Light*$^{-/-}$ mice. (**c**) Circulating cytokine levels in all four mouse groups. (**d**) Mac3^{+} area content in percentage of extravascular tissue in AngII–treatment in *Apoe*$^{-/-}$ and *Apoe*$^{-/-}$*Light*$^{-/-}$ mice. (**e**) CD3^{+} cells in the extravascular tissue in all four groups of mice. Data are shown as bars with individual data points and mean ± SEM. Statistical analysis was done using two-way ANOVA followed by Tukey's multiple comparison test. *$p \leq 0.05$ and **$p < 0.01$, and ***$p < 0.001$.

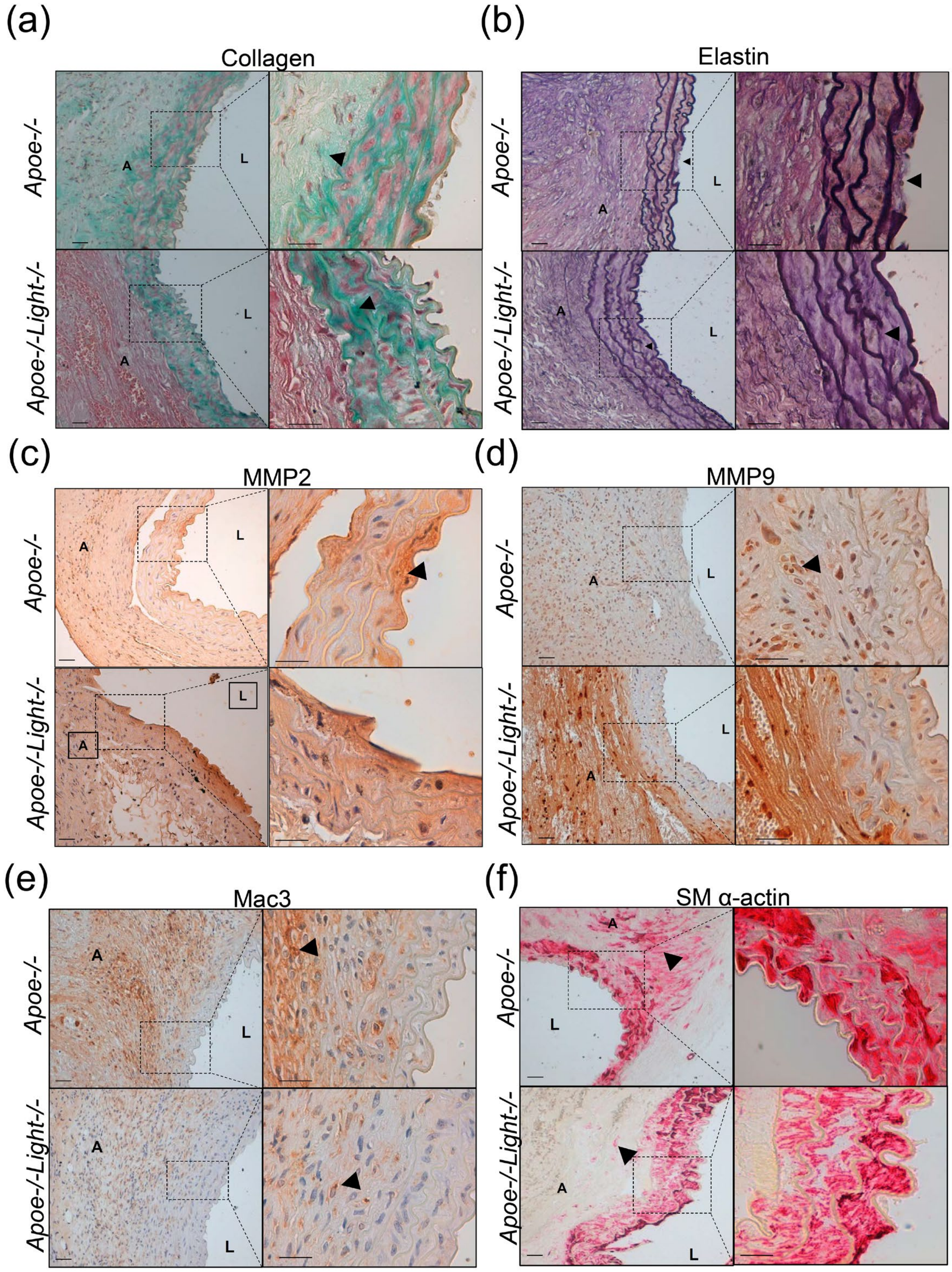

Figure S3. Representative images of stainings in AngII–treated *Apoe*$^{-/-}$ and *Apoe*$^{-/-}$*Light*$^{-/-}$ mice. Representative images and their magnifications of (**a**) collagen, (**b**) elastin, (**c**) MMP2, (**d**) MMP9, (**e**) Mac3 and (**f**) SM α-actin stainings in aneurysm cross-sections. Dashed squares limit the magnified area. Scale bar: 50 µm. Black triangles point to positive stainings and elastic fibers degradation. L: lumen and A: aneurysm.

Table S1. Sequences of the primers used to amplify human genes.

Gene	Forward Primer (5′→3′)	Reverse Primer (5′→3′)
ACTA2	AAAAGACAGCTACGTGGGTGA	GCCATGTTCTATCGGGTACTTC
BMP2	ACCCGCTGTCTTCTAGCGT	TTTCAGGCCGAACATGCTGAG
CKIT	CGTTCTGCTCCTACTGCTTCG	CCCACGCGGACTATTAAGTCT
GADPH	ACCACAGTCCATGCCATCAC	TCCACCACCCTGTTGCTGTA
KLF4	CCCACATGAAGCGACTTCCC	CAGGTCCAGGAGATCGTTGAA
LIGHT	ACGGCCCTCAGTGTTTGTG	TGTCTCCGGTGGCTTCGT
LTBR	GAGGGACCCAATCCTGTAGCT	TGTACCAAGTCAGGGAAGTATGGA
OCT4	CTG GGT TGA TCC TCG GAC CT	CCATCGGAGTTGCTCTCCA
OPN	CTCCATTGACTCGAACGACTC	CAGGTCTGCGAAACTTCTTAGAT
SCA1	TCGGTGGAGCTTGGTTTACAA	GGGAGGACCCAATGAACTGG
SOX9	AGCGAACGCACATCAAGAC	CTGTAGGCGATCTGTTGGGG

Table S2. Sequences of the primers used to amplify murine genes.

Gene	Forward Primer (5′→3′)	Reverse Primer (5′→3′)
Acta2	GTCCCAGACATCAGGGAGTAA	TCGGATACTTCAGCGTCAGGA
Bmp2	GGGACCCGCTGTCTTCTAGT	TCAACTCAAATTCGCTGAGGAC
Cd3	ATCGGTGGAACACTTTCTGG	GCACGTCAACTCTACACTGGT
Ckit	GCCACGTCTCAGCCATCTG	GTCGCCAGCTTCAACTATTAACT
Col1a1	GCTCCTCTTAGGGGCCACT	CCACGTCTCACCATTGGGG
Ccl19	GGGGTGCTAATGATGCGGAA	CCTTAGTGTGGTGAACACAACA
Ccl20	GCCTCTCGTACATACAGACGC	CCAGTTCTGCTTTGGATCAGC
Cxcl13	GGCCACGGTATTCTGGAAGC	GGGCGTAACTTGAATCCGATCTA
Cyclophilin	TGGAGAGCACCAAGACAGACA	TGCCGGAGTCGACAATGAT
Foxp3	GCCAACGCCCCAACAAG	CCCCGCCCACCTTTTCT
Gata3	GGTGGACGTACTTTTTAACATCGA	CGTAGCCCTGACGGAGTTTC
Klf4	GTGCCCCGACTAACCGTTG	GTCGTTGAACTCCTCGGTCT
Oct4	GGCTTCAGACTTCGCCTCC	AACCTGAGGTCCACAGTATGC
Opn	AGCAAGAAACTCTTCCAAGCA	GTGAGATTCGTCAGATTCATCCG
Rorc	GACCCACACCTCACAAATTGA	AGTAGGCCACATTACACTGCT
Sca1	AGGAGGCAGCAGTTATTGTGG	CGTTGACCTTAGTACCCAGGA
Sox2	GCGGAGTGGAAACTTTTGTCC	CGGGAAGCGTGTACTTATCCTT
Sox9	AGTACCCGCATCTGCACAAC	ACGAAGGGTCTCTTCTCGCT
Tbet	GCCAGGGAACCGCTTATATG	AACTTCCTGGCGCATCCA
Tgfb1	CTCCCGTGGCTTCTAGTGC	GCCTTAGTTTGGACAGGATCTG

References

1. Herrero-Cervera, A.; Vinué, Ángela; Burks, D.J.; González-Navarro, H. Genetic inactivation of the LIGHT (TNFSF14) cytokine in mice restores glucose homeostasis and diminishes hepatic steatosis. *Diabetol.* **2019**, *62*, 2143–2157, doi:10.1007/s00125-019-4962-6.
2. Daugherty, A.; Rateri, D.; Hong, L.; Balakrishnan, A. Measuring Blood Pressure in Mice using Volume Pressure Recording, a Tail-cuff Method. *J. Vis. Exp.* **2009**, e1291, doi:10.3791/1291.
3. Martorell, S.; Hueso, L.; Gonzalez-Navarro, H.; Collado, A.; Sanz, M.-J.; Piqueras, L. Vitamin D Receptor Activation Reduces Angiotensin-II–Induced Dissecting Abdominal Aortic Aneurysm in Apolipoprotein E–Knockout Mice. *Arter. Thromb. Vasc. Biol.* **2016**, *36*, 1587–1597, doi:10.1161/atvbaha.116.307530.
4. Ortega, R.; Collado, A.; Selles, F.; Gonzalez-Navarro, H.; Sanz, M.-J.; Real, J.T.; Piqueras, L. SGLT-2 (Sodium-Glucose Cotransporter 2) Inhibition Reduces Ang II (Angiotensin II)–Induced Dissecting Abdominal Aortic Aneurysm in ApoE (Apolipoprotein E) Knockout Mice. *Arter. Thromb. Vasc. Biol.* **2019**, *39*, 1614–1628, doi:10.1161/atvbaha.119.312659.
5. Hadi, T.; Boytard, L.; Silvestro, M.; Alebrahim, D.; Jacob, S.; Feinstein, J.; Barone, K.; Spiro, W.; Hutchison, S.; Simon, R.; et al. Macrophage-derived netrin-1 promotes abdominal aortic aneurysm formation by activating MMP3 in vascular smooth muscle cells. *Nat. Commun.* **2018**, *9*, 1–16, doi:10.1038/s41467-018-07495-1.

6. Vinué, Ángela; Martínez-Hervás, S.; Herrero-Cervera, A.; Sánchez-García, V.; Andrés-Blasco, I.; Piqueras, L.; Sanz, M.J.; Real, J.T.; Ascaso, J.F.; Burks, D.J.; et al. Changes in CDKN2A/2B expression associate with T-cell phenotype modulation in atherosclerosis and type 2 diabetes mellitus. *Transl. Res.* **2019**, *203*, 31–48, doi:10.1016/j.trsl.2018.08.003.
7. Mahnke, Y.D.; Beddall, M.H.; Roederer, M. OMIP-017: Human CD4+helper T-cell subsets including follicular helper cells. *Cytom. Part A* **2013**, *83*, 439–440, doi:10.1002/cyto.a.22269.
8. Qin, Y.; Cao, X.; Guo, J.; Zhang, Y.; Pan, L.; Zhang, H.; Li, H.; Tang, C.; Du, J.; Shi, G.-P. Deficiency of cathepsin S attenuates angiotensin II-induced abdominal aortic aneurysm formation in apolipoprotein E-deficient mice. *Cardiovasc. Res.* **2012**, *96*, 401–410, doi:10.1093/cvr/cvs263.
9. Martínez-Hervás, S.; Vinué, Ángela; Núñez, L.; Andrés-Blasco, I.; Piqueras, L.; Real, J.T.; Ascaso, J.F.; Burks, D.J.; Sanz, M.J.; González-Navarro, H. Insulin resistance aggravates atherosclerosis by reducing vascular smooth muscle cell survival and increasing CX3CL1/CX3CR1 axis. *Cardiovasc. Res.* **2014**, *103*, 324–336, doi:10.1093/cvr/cvu115.
